# Climate change and health in Pacific island states

**DOI:** 10.2471/BLT.15.166199

**Published:** 2015-12-01

**Authors:** Rokho Kim, Anthony Costello, Diarmid Campbell-Lendrum

**Affiliations:** a Division of Pacific Technical Support, World Health Organization Regional Office for the Western Pacific, Level 4, Provident Plaza One, Downtown Boulevard, 33 Ellery Street, Suva, Fiji.; b Department of Maternal, Newborn, Child and Adolescent Health, World Health Organization, Geneva.; c Department of Public Health, Environment and Social Determinants of Health, World Health Organization, Geneva.

The health and well-being of human populations – and the very existence of low-lying island states – depend on an ambitious outcome at the 21st Conference of the Parties to the United Nations Framework Convention on Climate Change, (Paris, 30 November to 11 December 2015). Unless the world dramatically reduces emissions of greenhouse gases, especially carbon dioxide from burning of fossil fuels, it is estimated that global average temperature will increase by 2.6–4.8 °C and sea level will rise by up to a metre around the end of this century.^1^ These changes would have major health impacts.

By the 2030s – now less than 15 years away – approximately 250 000 additional deaths are projected to occur every year from malnutrition, malaria, diarrhoea and heat stress attributable to climate change.^2^ Climate change will disproportionally affect small island states, because of their relatively small land area, high population density and dependence on local ecosystems for subsistence. Rising sea levels threaten to make low-lying island nations such as Kiribati, the Marshall Islands and Tuvalu uninhabitable. Understandably, Pacific island leaders have called for increases in global average temperatures to be limited to less than 1.5 °C.^3^

Pacific island countries are confronted with a triple burden of noncommunicable diseases, infectious diseases and climate change impacts. In some Pacific island states, mortality rates from noncommunicable diseases are already among the highest in the world. Since 2012, there have been over 40 large infectious disease outbreaks in the region: most were caused by climate-sensitive diseases such as dengue, chikungunya and Zika virus infections.^4^ There have also been several record-breaking extreme climate events in the Pacific recently. Extreme events often damage or destroy health facilities, disrupting essential health services when they are needed most urgently. For example, in Vanuatu, cyclone Pam seriously damaged many health facilities: two hospitals, 19 health centres and 50 dispensaries in 22 affected islands. In the most affected province, 21 of 24 health facilities were damaged. The economic impact was estimated to be more than 60% loss of gross domestic product.^5^

The World Health Organization (WHO) has been working with ministries of health and other stakeholders of Pacific island countries to build climate resilient health systems. Although a disease-specific approach is useful initially, in the long run a comprehensive intersectoral programme is required. As recommended by WHO,^6^ this programme should address (i) health governance and policies tackling climate risks; (ii) health information, integrated surveillance and climate early warning systems; and (iii) preventive and curative services (water and sanitation, pest and vector control, food safety, disaster risk management for health, safe and environmentally sustainable health facilities, vaccination and child health services).^7^

In the past five years, WHO has assisted thirteen Pacific island countries to develop national climate change and health vulnerability and impact assessments to guide health system adaptation plans. These assessments highlighted common climate risks such as direct impacts of extreme weather events (e.g. drowning and injuries), waterborne and vector-borne diseases.^8^ In Fiji, where a recent outbreak of dengue fever affected over 20 000 people, dengue, leptospirosis, typhoid fever and diarrhoea were targeted as priority climate-sensitive diseases.^9^ Fiji has produced a strategic action plan for climate change and health for 2016–2020, including the development of early warning and response in vulnerable communities. However, as for other small island developing states, implementation will be a huge challenge. The health sector has received less than 1.5% of the multilateral funds for climate change adaptation distributed to date.^10^

The international response to climate change is a major opportunity to improve global health. For the first time, international commitments for reductions in carbon dioxide emissions, if fully implemented, would have a meaningful impact on global temperature rise.^11^ The Paris climate pact, if sufficiently ambitious and effective, would be considered not only a major turning point in climate change policy, but also a far-reaching public health treaty to protect and promote the health and well-being of vulnerable populations, particularly those of small island states.^12^
